# The Impact of 40 Years of Data Collection: The Victoria Cerebral Palsy Register

**DOI:** 10.3390/children13081065

**Published:** 2026-08-10

**Authors:** Dinah S. Reddihough, Gina Hinwood, Angela Guzys, Erich Rutz, Susan M. Reid

**Affiliations:** 1 Neurodisability and Rehabilitation, Murdoch Children’s Research Institute, Parkville, VIC 3052, Australia; gina.hinwood@mcri.edu.au (G.H.); angela.guzys@mcri.edu.au (A.G.); sue.reid@mcri.edu.au (S.M.R.); 2 Department of Paediatrics, University of Melbourne, Parkville, VIC 3052, Australia; erich.rutz@rch.org.au; 3 Neurodevelopment and Disability, The Royal Children’s Hospital, Parkville, VIC 3052, Australia; 4 Victorian Paediatric Rehabilitation Service, Monash Children’s Hospital, Clayton, VIC 3168, Australia; 5 Department of Perinatal Medicine, The Mercy Hospital for Women, Heidelberg, VIC 3084, Australia; 6 Department of Orthopaedics, The Royal Children’s Hospital, Parkville, VIC 3052, Australia; 7 Orthopaedics, Clinical Sciences, Murdoch Children’s Research Institute, Parkville, VIC 3052, Australia

**Keywords:** cerebral palsy, Victorian Cerebral Palsy Register, epidemiology, population surveillance

## Abstract

**Highlights:**

**What are the main findings?**

- Registers are important in the understanding and management of cerebral palsy.
- They become more useful if they can be sustained over extended periods of time.

**What are the implications of the main findings?**

- The Victorian Cerebral Palsy Register has generated important information about epidemiology, risk factors and causation.
- It has provided an understanding of the many associated problems and has enabled further research to improve the health and wellbeing of children with cerebral palsy.

**Abstract:**

Background: The Victorian Cerebral Palsy Register (VCPR) project collects information on all individuals born or living in Victoria since 1970. It is one of the largest cerebral palsy (CP) registers internationally, with over 6800 participants. Methods: This paper will explain how the VCPR was established, how data are collected and the main outcomes from its use over an extended period of time. Results: The knowledge generated through the VCPR has contributed to information about epidemiology including trends in prevalence over time, and rates and causes of death; the VCPR has been used to investigate causal pathways and potential avenues for prevention or amelioration. It has been valuable in identifying cohorts for multidisciplinary studies resulting in improved understanding of the management of associated problems. The VCPR has also been used in the development of measurement tools and has enabled data linkage studies contributing to knowledge about CP. Conclusion: The Register not only provides an efficient means of identifying eligible cohorts, but the population basis of the VCPR provides the ability to assess the generalisability of research cohorts and a means of adjusting for selection bias and potential misinterpretation of study results. The project has gained international recognition for the knowledge generated on prevalence, risk factors, mortality, clinical profiles, neuroimaging patterns, health service use, assessment tools, participation, epidemiology in paediatric orthopaedics, and quality of life. It has underpinned significant improvements in clinical care for children with CP by enabling researchers from diverse disciplines to complete 157 studies.

## 1. Introduction

Cerebral palsy (CP) is an early-onset lifelong neurodevelopmental condition characterised by limitations in activity due to impaired development of movement and posture and commonly associated with abnormalities of muscle tone characterised clinically as spasticity, dystonia, choreoathetosis and/or ataxia. It is attributed to malformation of, or injury to, the foetal or infant brain that is not degenerative, although the clinical and functional manifestations may change with age [1].

People with CP contend with major musculoskeletal impacts: one in three are unable to walk independently, and mobility declines during adulthood [2]. Many have co-existing intellectual, vision, hearing and communication disabilities [3]. Epilepsy is common (two in five) and impacts life and function due to both the seizure disorder and the medications used to treat it [3]. Critically, three in four have ongoing pain and fatigue [4,5]. Recent research has demonstrated a high risk of preventable non-communicable diseases, such as diabetes, heart disease and stroke [6], and preventable mental health problems, including mood and anxiety disorders [7]. People with severe forms of CP may experience poor health which can impact wellbeing and quality of life and markedly reduces life expectancy.

In a systematic review, the birth prevalence of CP in the 1980s and 1990s was reported as 2.1 per 1000 live births [8]. In a more recent systematic review, the prevalence in high-income countries has declined to 1.6 per 1000 live births. From limited data available from low- and middle-income countries, rates remain high at 3.4 per 1000 live births [9]. Understanding more about the epidemiology of CP is essential to our quest to reduce rates globally, given the high burden of this condition. CP registers have long been considered vital resources to describe and monitor changes over time in CP populations in terms of prevalence, clinical presentations, causation, and length of survival [10]. The number of CP registers has increased over time. A recent survey identified 57 different CP registers, including many new registers in low-and middle-income countries [11].

Following in the footsteps of the successful Western Australian CP Register [12], data collection for a population-based Victorian CP Register (VCPR) began in 1986, collecting data prospectively and retrospectively on individuals born in Victoria from 1970 onwards. Although predominantly having an epidemiological focus, requests were increasingly received to use register data to support other research, given its ability to facilitate efficient study recruitment across a population-based cohort. This has provided the opportunity to do more than epidemiological research, and to work with multidisciplinary teams across Australia and internationally to embark on studies of clinical importance. In 2007, the decision was made to register non-Victorian-born cases receiving health services in Victoria to allow equity of access to research opportunities.

The aim of this paper is to describe the methods, outputs, and impact of the VCPR over 40 years, in particular to describe:

1. How it has contributed to knowledge creation about trends in prevalence and length of survival;
2. How it has been used to understand causation and identify potential avenues for prevention or amelioration of the condition;
3. Its value in identifying cohorts for studies across multiple disciplines;
4. How the use of cohorts has improved understanding of the management of associated problems;
5. Use in the development of measurement tools;
6. Contribution to the Australian Cerebral Palsy Register;
7. Use in data linkage studies.

To our knowledge, the overall outcomes of a CP register have not been previously reported, but only individual projects resulting from them. Most publications report on the epidemiology in their region of origin, and in some cases, cohorts have been selected to examine specific clinical features such as pain [13]. Given the time, resources and commitment involved in sustaining a register over an extended period, our goal is to provide an audit of what has been achieved and the impact of data collection, including the scientific outcomes, over an extended period of time. This report may also be useful for researchers planning to establish new registers.

## 2. Materials and Methods

### 2.1. Study Design and Setting

This publication reports on epidemiological studies undertaken from data extracted from the VCPR. In addition, examples are provided of how various cohorts from the VCPR have been used over an extended period, to undertake different studies to understand more about CP, to improve management and to develop measurement tools.

The co-located clinical and academic settings for the VCPR are The Royal Children’s Hospital and Murdoch Children’s Research Institute in Melbourne, Australia.

### 2.2. Ethics

Ethics approval from The Royal Children’s Hospital Human Research Ethics Committee (HREC) for the VCPR has been in place since 1991 with the latest approved protocol version 12 dated 9 June 2022. VCPR studies are conducted in accordance with the Declaration of Helsinki. Specific site governance approvals are in place from the Monash Health HREC and the Mercy Health HREC. Ethical review and approval were waived for this study due to secondary data analysis of data extracted from the VCPR.

### 2.3. Case Ascertainment and Consent

Given that the VCPR was established almost 40 years ago, the methods of data collection have changed over the years. When the VCPR was started, research assistants manually reviewed hard copy patient case histories. Currently, potential new cases are mainly identified through reporting algorithms within the electronic medical records at the two tertiary-level paediatric institutions in Victoria. A minority are identified via clinician, family or self-referral, or from interstate registries. This algorithm identifies children with a CP diagnosis amongst inpatient admissions and relevant outpatient clinic appointments over the previous month. The lists are uploaded monthly into a Microsoft Access database, duplicate records are removed, and the electronic medical records of new cases are checked against the eligibility criteria (see below). A strategy is in place to re-check the records when eligibility is at first unclear. Once the diagnosis of CP is verified, families are contacted by mail or email for consent for their child’s details to be added to the VCPR. Prior to 2015, consent was optional, but an opt-out approach has been in operation since 2015. For families that opt out, name, sex, date of birth, death status and date of death (if relevant), place of birth, and diagnosis are recorded. Registrants are also asked to consent to (1) periodic transfer of non-identifiable data to the Australian CP Register, (2) contact from Register staff about new research opportunities, and (3) data linkage through specialised data linkage units. If families choose to opt out, a minimum dataset is retained for ‘counting’ purposes.

### 2.4. Case Definition and Eligibility Criteria

The most widely accepted definitions of CP from the published literature have changed over the life of the VCPR. We have consistently applied the following criteria that are implied in all definitions: (1) it is a permanent, but not unchanging condition; (2) it involves a disorder of movement and/or posture that limits function; (3) it is due to a non-progressive interference/lesion/abnormality; and (4) the interference/lesion/abnormality originates in the developing/immature brain. Data are included if the person with CP was born in Victoria from 1970 onwards or lived and received health services in Victoria during those birth years, and if the brain injury occurred before the age of 2 years.

### 2.5. Data Sources

Data for the VCPR are mainly collected from the child’s electronic medical record at the two tertiary-level paediatric institutions in Victoria. Additional maternal demographic data and information pertaining to the pregnancy, birth, and neonatal period are derived from the Birthing Outcomes System at two hospitals with tertiary maternity services, as well as from birth and neonatal discharge summaries, and from parental surveys.

### 2.6. Confirmation of Diagnosis and Data Updates

Acknowledging that diagnosis is not always straightforward, particularly in young children, the diagnosis is confirmed or refuted at the age of five years [14]. At that time, the data on each child are also updated. This is the standard for Australian and international CP registries, both to provide consistency for comparisons and because earlier data are not always complete or reliable. Further data updates for the VCPR are undertaken at ages 10, 15 and 20 years, if resources are available.

### 2.7. Data Extraction and Evaluation Procedures

Data for the VCPR are extracted by two experienced research assistants and reviewed by a senior research officer for quality assurance purposes. For the various studies described, researchers including senior clinicians, allied health professionals and doctoral and postdoctoral students have been involved.

### 2.8. Data Collection Period

The work reported in this paper covers the period 1970–2024. Early on in the course of the project, the number of participants was small and it was not possible to undertake meaningful studies. Most of the work described in this publication covers the cohort born 1995–2024.

### 2.9. Types of Data Collected

The information collected about each child covers seven broad areas: (1) demographics, e.g., date of birth, postcode; (2) antenatal and perinatal data, e.g., birth weight, gestation, condition at birth, need for intensive care in the neonatal period; (3) congenital anomalies classified using the European Surveillance of Congenital Anomalies (EUROCAT), e.g., brain and heart malformation [15] classification and definitions; (4) aetiology and contributing factors, including genetic investigations; (5) magnetic resonance imaging (MRI) findings classified using the Neonatal Neuroimaging Classification System (NNICS) [16] and the MRI Classification System (MRICS) [17] which provides information about the nature, timing and severity of the brain injury in CP; (6) the nature and severity of the motor disorder (the latter using the Gross Motor Function Classification System (GMFCS) [18] (categorises the movement abilities of individuals with CP into five levels) and Manual Ability Classification System (MACS) (describes how children with CP use their hands to manipulate objects in everyday life [19])); and (7) the presence of co-existing impairments and conditions, e.g., hearing and visual impairment, epilepsy. A total of 438 variables is collected for each participant.

### 2.10. Analytical Approaches to Data Use

For epidemiological studies, cohorts are selected according to birth year with the study period defined. In other research involving the Register, careful inclusion and exclusion criteria are defined in the study protocols which are written prior to study commencement.

### 2.11. Upload to the National Dataset

Every two years, an agreed minimum dataset is uploaded to the Australian CP Register. Amalgamated data from all children born in each of the six states and two territories of Australia provide the basis for national reports every 2–4 years [20].

## 3. Results

### 3.1. Cumulative Case Ascertainment

**Figure 1:**
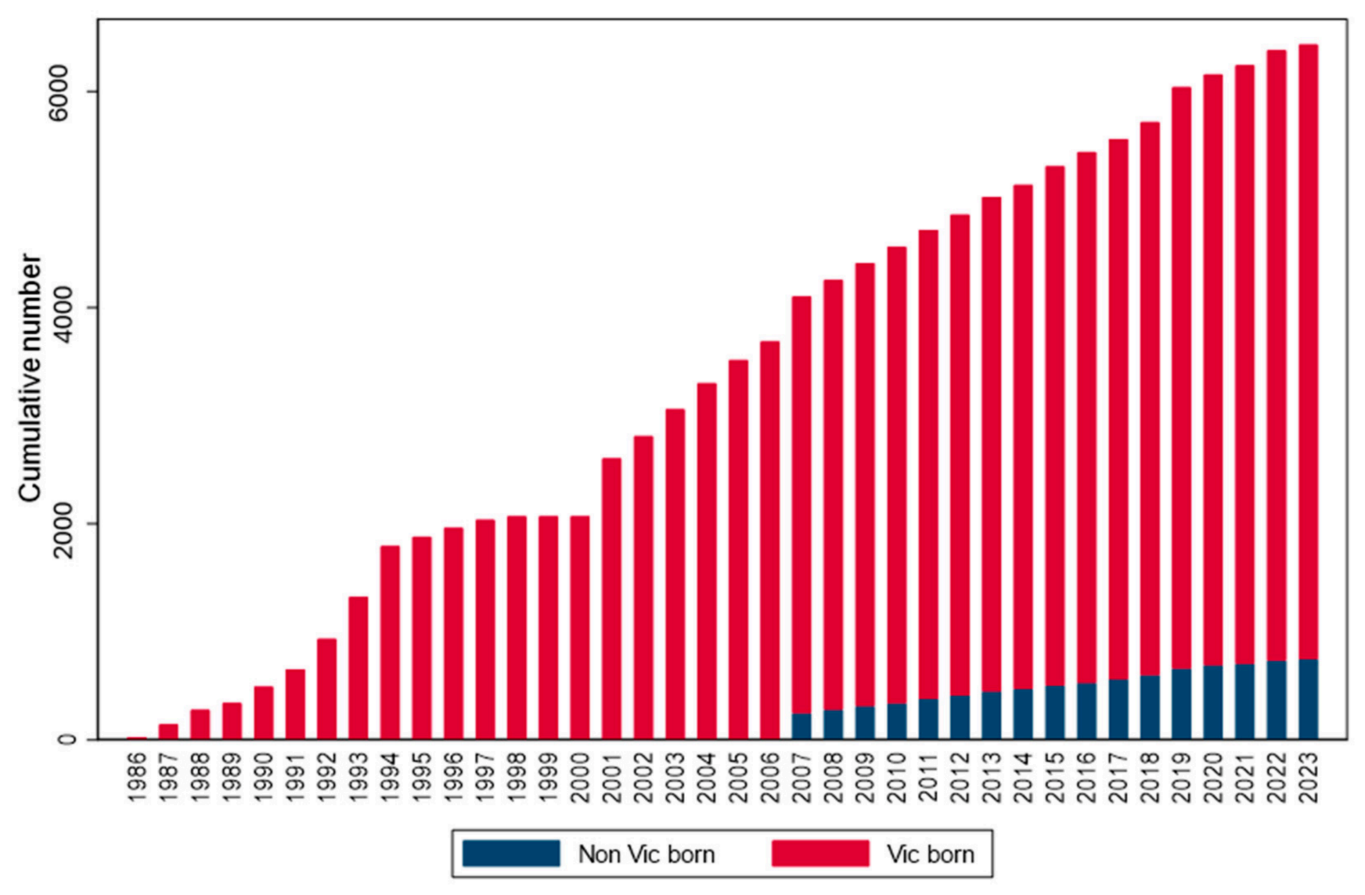
Cumulative ascertainment of CP cases in Victoria between 1986 and 2023 showing steady growth when funding was available and the effect of adding in people born outside Victoria.

Case ascertainment reached 6500 at the end of 2023 (Figure 1). The large jump upwards in 2001 was due to the receipt of a grant and appointment of two research assistants.

### 3.2. Contribution to Knowledge Creation About Trends in Prevalence and Length of Survival

#### 3.2.1. Trends in Prevalence

**Figure 2:**
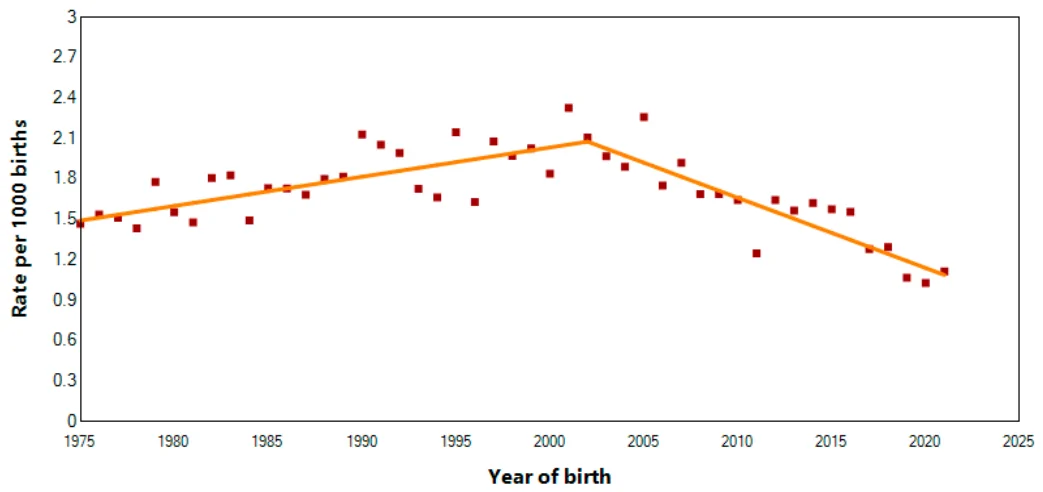
Joinpoint regression model for rates of CP per 1000 births in Victoria between 1975 and 2022 showing increasing rates over the 1970s, 80s and 90s and decreasing rates since 2002.

After a period of rising rates of CP in Victoria over the 1980s and 1990s, a trend believed to be due to improved survival of extremely preterm infants (<28 weeks’ gestation), in 2016, we were finally able to report declines in rates and severity of CP over the 2000s at all birth gestations [21]. Recently, we confirmed continuation of this decline in prevalence in a 2000–2019 birth cohort (published online, Figure 2). Over the same 20-year period, we also observed a 45% rate reduction specifically for severe CP (GMFCS levels IV and V; paper under review).

#### 3.2.2. Length of Survival and Causes of Death

In a VCPR cohort of individuals born between 1970 and 2004, the probability of survival to 40 years was estimated to be 80%, with the highest risk of early mortality seen in the group with greater clinical complexity. In that study, no temporal improvement in survival was observed (unadjusted hazard ratio 1.00 [95% CI 0.99, 1.01]) [22]. In a more recent study of persons with CP born between 1970 and 2012, survival analysis revealed a little improvement in the probability of survival to 40 years (83%) and a trend towards delayed mortality, with 35% improvement in the probability of survival to 15 years for those born in the 2000s compared to the 1970s [23]. Respiratory conditions were the predominant causes of death [22].

### 3.3. Use of VCPR Data to Understand Causation and Identify Potential Avenues for Prevention or Amelioration of the Condition

From a patho-aetiological perspective, CP is a heterogeneous condition. Many antecedent factors have been investigated for their contribution to an increased risk of developing CP. Thrombophilic mutations [24], assisted reproductive technologies [25], biological sex [26], epigenetic biomarkers [27], and maternal serum biomarkers obtained during the first or second trimester of pregnancy [28,29] have all been shown to be associated, to varying degrees, with specific subgroups of CP cases in Victoria.

Neuroimaging, particularly magnetic resonance imaging (MRI), is of paramount importance in identifying the likely mechanism and timing of brain injury in CP. Using the NNICS and MRICS, the VCPR has classified the neuroimaging of children in all birth cohorts from 1999 onwards, the period over which MRI was more widely used. The MRICS consists of (A) brain maldevelopment, (B) predominant white matter injury, (C) predominant grey matter injury, (D) miscellaneous, and (E) normal MRI [17]. The VCPR has been at the forefront of work on MRI findings in CP. Using Victoria data, we have associated risk factors with specific patterns of brain pathology [30,31], and correlated neuroimaging patterns with clinical phenotypes [32].

The addition of neuroimaging data to existing VCPR data on factors relevant to the pregnancy and perinatal period, clinical phenotype, presence of congenital anomalies, and results of investigations, including genetics, has facilitated the development of a new causal pathway classification. Because CP is such a heterogeneous condition, there are many advantages to be gained from taking a more focused causal pathway approach. It enables better understanding of timing and mechanisms of brain injury, the pathways in which changes in practice are driving changes in rates of CP and in the complexity of the condition, and where preventative efforts might best be pursued. Several areas of research have incorporated our causal pathway classification, described below.

In a cohort comprising 1348 individuals with CP born from 35 gestational weeks, an estimated 57% of the cohort had a complicated neonatal period. Timing of brain injury was antenatal in 57% and perinatal in 41%. Our data showed a decrease in the relative proportions of perinatal timing of brain injury over a period when the rates of CP in live births at term decreased [33]. These data suggest that advances in perinatal and neonatal care have had an impact on neurological morbidity.

Another study furthered our understanding of the contribution of congenital anomalies to development of CP. In a large population cohort of 2238 persons born in Victoria between 1999 and 2017, we reported that 23% had major congenital anomalies and 17% of the cohort had anomalies that potentially contributed to the development of CP, with the remaining 7% unlikely to be pathogenic. Of those with congenital abnormalities, the majority were cerebral, present in 14% of the cohort, and involved developmental causal pathways. Only 3% (predominantly severe cardiac anomalies) were related to destructive brain insults [34].

In the third study, data from 2303 children born in Victoria between 2000 and 2019 and trends in the birth cohort prevalence of CP per 100,000 livebirths were studied for well-defined subgroups based on likely causal pathway, birth condition, and neonatal course. The steepest declines in rates of CP in Victoria were for causal subgroups involving perinatal cerebral insults and significant neonatal complications (annual percent change −5.98 [95% CI −8.46, −3.44]), suggesting that advances in perinatal care may be driving the decreasing rates. There was also a decline in rates of CP associated with birth before 35 weeks’ gestation and perinatal insults commonly seen in preterm neonates (annual percent change −3.12 [95% CI −4.63, −1.58]). Evidence was lacking for sustained decreases in CP rates for subgroups of children with early developmental causes, perinatal arterial ischaemic strokes, predominant white matter injury patterns in association with birth from 35 weeks, and postneonatal causes [35].

### 3.4. The Value of the VCPR in Identifying Cohorts for Studies Across Multiple Disciplines

The VCPR has been used to determine the frequency of associated problems in CP. Four examples are provided. In a population-based study of hearing loss in children with CP (mean age 8 years and 1 month (SD 1 year 7months range 5 years 1month to 11 years)), 3–4% had a severe-profound hearing loss [36].

In a further study, we established that language impairment was common in 5-year-old and 6-year-old children with CP, affecting three out of five children. Twenty-four per cent of a cohort of 84 children were non-verbal. Co-occurring receptive and expressive language impairment occurred in 44% [37]. In a cohort comprising 1141 children with CP to establish the co-occurrence of intellectual disability in CP, 12% had missing data about cognitive abilities, whilst 45% of the remaining 1005 children had a diagnosis of intellectual disability [38]. Drooling is a significant problem for individuals with CP, impeding integration into school and community settings. A total of 385 children of mean age 10 years 9 months (SD 1 year 7 months, range 8–14 years) were studied. After adjustment for topographical pattern of motor impairment and GMFCS level, 40% were reported to have experienced drooling between 4 years of age and the time of completing the questionnaire. A significantly higher prevalence of drooling was found in children with poor gross motor function and in those with more severe presentations of CP, including poor head control, difficulty with eating, and inability to sustain lip closure (*p* < 0.001 for each). Drooling was shown to be significantly associated with both intellectual disability and epilepsy in this group of children (*p* < 0.001 for both) [39].

### 3.5. The Use of Cohorts Has Improved Understanding of the Management of Associated Problems

Three examples are given.

#### 3.5.1. Epilepsy

Using a cohort of 256 children with prenatal or perinatal vascular injuries on MRI selected from the VCPR, 93 (36%) had one or more febrile or afebrile seizures beyond the neonatal period and 87 (34%) had epilepsy [40]. Fifty-six (60%) of these children had electroclinical features of a self-limited focal epilepsy of childhood; of the 93 children with seizures, at last follow-up (mean age 15 years), 61/91 (67%) had not had a seizure in >2 years [40]. Compared to typically developing children with epilepsy, self-limited focal epilepsy-variant occurred much more commonly in children with CP.

#### 3.5.2. Hip Displacement and Other Orthopaedic Conditions

In the early 1990s, a cohort of children born 1990–1992 was selected from the VCPR. Using the GMFCS, we established a linear relationship between hip displacement and level of motor function, with those at GMFCS V having the greatest risk (90%). The resulting publication has been cited on 456 occasions [41] and has formed the basis of hip surveillance guidelines, now used both in Australia and more widely. The same study cohort was followed up for epidemiological studies regarding scoliosis and musculoskeletal pathology in cerebral palsy [42,43,44,45].

#### 3.5.3. Saliva Control Surgery

Surgical procedures, known as sialodochoplasties, are often performed for the control of drooling. These may include major salivary gland excision, parasympathetic nerve section, and duct ligation and re-routing. Alterations in saliva amount, flow, and consistency occur following this procedure, and the resultant effect on dental homeostasis was investigated. In a controlled study, 19 children with CP following sialodochoplasty (surgery group) and 75 children with CP treated non-surgically (control group) were investigated. The surgical group had significantly more dental caries when compared with the control group [46]. Although no caries predictors were identified, alterations to the caries-protective role of saliva are considered the likely cause.

### 3.6. Using the Victorian CP Register to Develop Measurement Tools

Three examples are provided.

#### Quality of Life in Children with CP

The CP Quality of Life Questionnaire for Children (CP QOL-Child) was the first quality-of-life measure specifically for children with CP, designed to assess wellbeing rather than ill-being. It was developed using a cohort of 205 parents and 53 children from the VCPR [47]. CP QOL-Child, based on the International Classification of Function, was developed with international expertise and recognised the importance of obtaining the views of the child and primary caregivers in developing and completing the questionnaire. The starting age of 4 years was chosen to ensure that the child was old enough to have a clear diagnosis of CP. Children aged over 12 years were not included as it was considered that new issues, such as body image, school pressures, and employment would emerge during adolescence. Subsequently the VCPR was used to develop the CP Quality of Life—Teen. Questionnaires were returned by 112 primary caregivers, and 87 adolescents aged 12–18 years also completed the questionnaires [48].

The Quality of Life Inventory—Disability (QI-Disability) is a parent-reported outcome assessment of quality of life for children and adolescents with intellectual disability, suitable to use for children and adolescents aged 3 to 18 years and with adults who live with severe disability conditions [49]. Families were recruited from five databases, including the Victorian CP Register (those with CP and intellectual disability), to undertake both qualitative and quantitative studies.

The Victorian CP Register was used to recruit participants to develop the Melbourne Assessment of Unilateral Upper Limb Function, a discriminative and evaluative measure of upper limb function [50].

### 3.7. Contribution to the Australian Cerebral Palsy Register

The VCPR is a key contributor to the Australian Cerebral Palsy Register (ACPR). A widely circulated report on CP in Australia is compiled every 3 years [51,52,53]. VCPR data has also been used in numerous research projects undertaken by the ACPR including a study into cause-specific secular trends and prevention measures of postneonatally acquired CP [54], differences in birth prevalence by residential remoteness, prevalence and trends for Aboriginal and Torres Strait Islander children living with CP [55], and congenital anomalies in children with pre- or perinatally acquired CP [56,57].

### 3.8. Use in Data Linkage Studies

After linkage with admissions data from the two tertiary paediatric hospitals [58], Victorian Data Linkages were used to link VCPR data with the Victorian Admitted Episodes Dataset, a dataset of hospital admissions for the entire state. These data showed that 80% of the CP cohort (n = 1401) had at least one admission over an 8-year period. Their admissions accounted for 1.5% of all admissions in their age group and were comparatively more expensive. Increased complexity of CP was associated with multiple admissions with a large proportion attributable to respiratory illness. Similar data linkages were performed for emergency department presentations. Children with CP accounted for 0.3% of all emergency department attendances. The number of annual presentations per 1000 children rose with increasing CP severity. Compared with presentations among the general population, higher proportions of presentations among the CP cohort were preceded by ambulance arrivals (27% vs. 8%), triaged as urgent (66% vs. 32%), and required hospital admission (38% vs. 12%) [59,60].

## 4. Discussion

Decisions to establish a register are not made lightly. During the initial period of establishment, there was little research that could be done. It was only after a period of time that the real value of the VCPR has become apparent, when large and diverse cohorts could be recruited, for epidemiological investigation, management and outcomes of people with CP. The VCPR has enabled 157 projects bringing together multiple disciplines, resulting in 180 publications and enabling 60 students to undertake higher degrees. The impact is therefore substantial and is particularly important for a heterogeneous condition with multiple associated conditions and morbidities. Although all of the work detailed in this paper has been reported previously as individual projects, no previous attempt has been made to summarise the work of the VCPR and its contribution over an extended period.

The VCPR has been responsible for changing practice. Each facet of the work undertaken has been significant. Our team was one of the first to show a decline in CP rates which was later confirmed by other groups [9,21,61].

CP prevalence in high-income countries has decreased by about 25% over the past 20 years but remains at about double that rate in low- and middle-income countries. Ongoing surveillance assists in measuring the efficacy of medical advances in obstetric and perinatal care in reducing the rates and severity of CP [9]. Epidemiological research has also assisted in planning services in Victoria, Australia [21]. Estimation of life expectancy has been used repeatedly in litigation cases. Our work on causal pathways has demonstrated where there has been a reduction in cases in those subgroups involving perinatal cerebral insults and in the subgroup associated with birth before 35 weeks’ gestation. Other groups have also shown declines in rates of CP in those born prematurely. In Europe, prevalence decreased between 2004 and 2010 for children born between 28 weeks’ and 36 weeks’ gestation, but the trend was less clear for those born before 28 weeks [62] and was not always consistent between countries [63].

Epilepsy, hip displacement and drooling are all significant problems with improvements in care made possible by the VCPR. The relatively good prognosis for epilepsy has also been confirmed in a Swedish cohort of 140 children in which 45% were seizure-free for at least one year by 13–16 years [64]. These epilepsy studies have revolutionised our understanding of seizure disorders in CP with important implications for treatment supporting earlier reduction and discontinuation of antiepileptic medication and providing information for counselling about prognosis. Hip surveillance guidelines have been pivotal in improving the care of children with CP as early detection of hip displacement reduces the incidence of painful dislocation [65]. Drooling impacts on many areas of life: clothes and equipment may be damaged, odour may be significant, but most importantly, it may impact on the child’s psychosocial wellbeing, causing poor self-esteem and social isolation. Our work has drawn attention to the frequency of the problem. Many studies are now available evaluating the outcomes of various treatments including pharmacotherapy and surgery including a recent systematic review [66]. To our knowledge, the impact that surgery may have on dental health has not been highlighted elsewhere.

Measures developed are used widely. The CP QOL-Child, QI-Disability and the Melbourne Assessment of Upper Limb Function are used widely in diverse studies internationally. CP QOL-Child has been translated into more than 20 languages and QI-Disability is employed as an outcome measure in clinical trials of therapeutics for rare diseases.

Assessment tools are pivotal for the care of children with CP, providing a foundation on which to tailor interventions and make informed decisions about treatment strategies [67]. The contribution from the VCPR to the creation of assessment tools has been significant. Data linkage studies have helped us to understand how health services are provided to children with CP and where there is opportunity for improvement.

Registers consume extensive ongoing resources in terms of time and funding but provide the ability to assess the generalisability of research cohorts and a means of adjusting for selection bias and potential misinterpretation of study results [68].

## 5. Limitations

The strengths of the VCPR are its longevity with a subsequent large number of registrants and the ability to recruit cohorts over time for multiple studies. There are some limitations. Most children with CP attend the two major paediatric hospitals, where most of the data is collected, at some stage during their childhood. Those with severe impairments are likely to attend very early in life. It is possible that some children with very mild impairments may not have a reason to visit a tertiary hospital and hence may not be included in the VCPR. We estimate that this would not be more than 5% of children with CP. It is also possible that the research team who scan the records could miss relevant data. However, they are all extremely experienced and have been involved in collecting data for the VCPR for many years.

## 6. Conclusions

The work associated with the VCPR has extended for over 40 years and has contributed substantially to knowledge about the epidemiology, risk factors, associated problems, management and quality of life. The VCPR has been able to demonstrate declining CP rates in Victoria, particularly in two cohorts of children, those born <35 weeks’ gestation and those with perinatal cerebral insults and significant neonatal complications, suggesting that advances in obstetric and neonatal care have made a significant contribution. Due to the population basis of our work, we have been able to demonstrate the rates of various comorbidities including hearing impairment and intellectual disability. The ability to recruit cohorts for specific research projects has enabled advances in clinical care, for example, hip surveillance guidelines.

Now that a significant proportion of people on the VCPR are adults, a unique opportunity is provided to study longer-term outcomes and address the health of this underserved population. This cohort often experiences severe physical and mental health challenges and generally there is a lack of appropriate services to cater for their needs. Considerably more research is required in this area [69].

Advocacy is needed to obtain resources to continue and expand the work. A greater understanding of the usefulness of the VCPR, by consumers, health professionals and others in the community, will be important to ensure that these resources are secured.

## Data Availability

The original contributions presented in this study are included in the article. Further inquiries can be directed to the corresponding author.

## Abbreviations

The following abbreviations are used in this manuscript:

VCPR	Victorian Cerebral Palsy Register
CP	Cerebral palsy
HREC	Human Research Ethics Committee
EUROCAT	European Surveillance of Congenital Anomalies
MRI	Magnetic resonance imaging
NNICS	Neonatal Neuroimaging Classification System
MRICS	Magnetic Resonance Imaging Classification System
GMFCS	Gross Motor Function Classification System
MACS	Manual Ability Classification System
CP QOL-Child	Cerebral Palsy Quality of Life Questionnaire for Children
QI-Disability	Quality of Life Inventory—Disability
